# Lipid Keratopathy: Histopathology, Major Differential Diagnoses and The Importance of Clinical Correlation

**DOI:** 10.3390/diagnostics13091628

**Published:** 2023-05-04

**Authors:** Nora Knez, Molly Walkenhorst, Mohammad Haeri

**Affiliations:** 1 Department of Pathology and Laboratory Medicine, The University of Kansas Medical Center, Kansas City, KS 66103, USA; noraknez6@gmail.com (N.K.); mwalkenhorst@kumc.edu (M.W.); 2 School of Medicine, University of Zagreb, 10000 Zagreb, Croatia

**Keywords:** lipid keratopathy, herpes zoster keratitis, corneal neovascularisation, vision loss, corneal arcus, Schnyder corneal dystrophy

## Abstract

Lipid keratopathy (LK) is a rare ophthalmological condition characterized by a progressive reduction in visual acuity caused by corneal opacification due to central lipid accumulation. LK is characterized by lipid deposits, cholesterol clefts, and neovascularization (NV) leading to disruption in corneal optical quality. LK classification includes a primary and secondary form which depend on pre-existing corneal or systemic disorders and the evidence of NV. Secondary LK is typically associated with a prior occurrence of herpetic infection, such as herpes zoster keratitis. Patients with LK usually present with progressive vision loss and dense cream-colored corneal opacification. Treatment modalities include conservative and surgical approaches focused on corneal NV elimination. When evaluating corneal lipidosis, it is crucial to consider a range of differential diagnoses, including corneal arcus, Schnyder corneal dystrophy, and other corneal deposit conditions. We report a case of a 62-year-old male with herpes zoster keratitis complicated with LK. He presented with painless progressive vision loss and corneal scarring, which raised suspicion about LK diagnosis. This paper emphasizes the importance of correlating clinical and histological findings for accurate LK diagnosis.

Lipid keratopathy (LK) is a rare ophthalmological condition characterized by a progressive and painless reduction in visual acuity caused by corneal opacification due to central lipid accumulation [1]. The typical histopathological characteristics of lipid keratopathy include lipid deposits, the formation of cholesterol clefts, and neovascularization (NV), all of which contribute to a reduction in the optical quality of the cornea [2].

The etiological LK categorization includes primary and secondary forms which are determined by pre-existing corneal or systemic disorders and the evidence of NV. Primary or idiopathic LK arises spontaneously, without preceding ocular pathology, and with lipid levels in reference ranges [3]. Secondary LK is associated with previous corneal inflammation, trauma, iatrogenic damage, or degeneration. Systemic diseases such as dyslipidemia can also be considered [2,4]. Corneal NV represents healing sequelae usually due to the inflammatory process [4]. Herpes zoster keratitis is also a significant cause of secondary LK due to inflammation, NV, ulceration, scarring, and subsequential lipid deposition [5].

The characteristic pathohistological features of LK are lipid deposits visualized by Oil Red O and Sudan Black B staining on frozen tissue [1,2]. Additionally, when frozen sections are unavailable, the only method to confirm the diagnoses are cholesterol clefts and NV visualization on H&E slides which are pathognomonic pathohistological findings in LK [1]. Other features include inflammatory foci beneath the epithelium with the destruction of the Bowman’s layer throughout the stroma down to the Descemet membrane. Stromal edema and basement membrane thickening can also be visualized [5].

Patients with LK usually present with progressive and painless deterioration in visual acuity and characteristic dense cream-colored opacification [2,4]. Symptoms of the underlying disease might also be present in patients with secondary LK [2].

Corneal lipid accumulation is not a specific feature of LK, and other ophthalmological disorders should be considered in patients with a progressive decrease in visual acuity and corneal opacification [2]. Corneal arcus, Schnyder corneal dystrophy (SCD), and other corneal deposit conditions (cystinosis, tyrosinemia, hyperuricemia, multiple myeloma, and monoclonal gammopathy) or drug corneal deposition (gold, chlorpromazine, chloroquine, and clofazimine) might be considered in the differential diagnoses [2]. Corneal arcus is the most common form of irreversible peripheral corneal lipidosis with preserved visual acuity [5,6]. Arcus senilis is the name of corneal arcus which occurs as part of the normal aging process and may be seen commonly in older individuals. Rarely, corneal arcus might be associated with underlying lipid disorders such as hypercholesterolemia and familial hyperlipidemia [2]. Corneal arcus is identified by a white or grey opaque ring surrounding the iris [7]. In contrast to LK, corneal arcus has minimal signs of inflammation, and the deposits are primarily extracellular [2]. SCD is a rare autosomal dominant disorder characterized by progressive bilateral corneal opacification caused by impaired lipid metabolism with subsequential corneal lipid deposits [8]. The main feature of SCD is corneal arcus development at an early age, typically during the first few decades of life [2]. SCD is not associated with inflammation or corneal NV [9]. It is noteworthy that systemic dyslipoproteinemias, including fish-eye disease (FED), Tangier disease (TD), and familial lecithin–cholesterol acyltransferase (LCAT) deficiency (FLD) may also lead to corneal lipid deposition [2].

We report the case of a 62-year-old male who presented with painless progressive vision loss of the right eye. In 2016, the patient presented with right eye corneal scarring and a minor decrease in vision with an acuity of 20/60. It was determined that these findings were likely complications resulting from a previous occurrence of ophthalmic herpes zoster infection in the patient’s right eye in 2015. In 2020, during a routine ophthalmological follow-up, the patient had significant worsening of his vision in the right eye with deterioration of visual acuity to 20/400 and further corneal scarring. The eye exam was consistent with zoster interstitial keratitis complicated by lipid keratopathy. The patient’s condition continued to worsen despite conservative treatment, and the patient elected to proceed with a right penetrating keratoplasty. Follwing the keratoplasty the corneal specimen was sent for histological assessment. The cornea demonstrated stromal edema, vacuolization, fibrosis with focal disruptions in the Bowman’s layer, and needle-shaped cholesterol deposits (clefts). Additionally, a thickened epithelial basement membrane and focal subepithelial pannus were noted. The endothelial cell layer was attenuated. Periodic acid–Schiff staining highlighted the mentioned pathological findings and stromal vessels, altogether confirming the diagnosis of lipid keratopathy. Additionally, the HSV1, trichrome, and Congo Red stains were negative. Although fresh frozen tissue for special staining with Oil Red O was unavailable, cholesterol deposits on the H&E slides provided sufficient evidence to confirm LK diagnosis (Figure 1).

Lipid keratopathy is a rare ophthalmological disorder that should be considered in the differential diagnosis of corneal opacification and progressive painless visual acuity decline. Correlating the clinical picture with the pathology is essential in diagnosing lipid keratopathy. It is important to determine if the patient has symptoms that could raise concern for secondary lipid keratopathy, as appropriate treatment of the underlying condition may improve the patient’s pathology and clinical outcome. Similar pathologies might be seen in corneal arcus, SCD, and other corneal deposit conditions, which must be included in the differential diagnoses of LK. In cases where neovascularization and inflammatory keratitis lead to secondary lipid keratopathy, the underlying cause is typically associated with a prior occurrence of herpetic infection, such as herpes zoster keratitis.

## Figures and Tables

**Figure 1:**
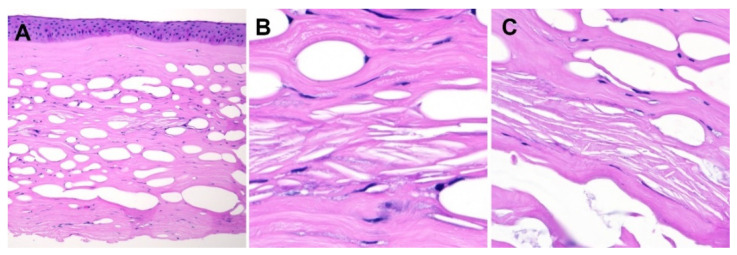
H&E images of the patient’s cornea. (**A**) The cornea shows mild stromal inflammation with needle-shape cholestorl deposits (clefts) shown at higher magnificaitons (**B**,**C**).

## Data Availability

Not applicable.
